# Callus Induction and Plant Regeneration from Immature Embryos of Sweet Sorghum (*Sorghum bicolor* Moench)

**DOI:** 10.3923/biotech.2018.12.18

**Published:** 2018-03-01

**Authors:** Rasha Adam Omer, Pauline Asami, Josephine Birungi

**Affiliations:** 1 Biosciences Eastern and Central Africa-International Livestock Research Institute (BecA-ILRI) Hub, P.O. Box 30709, Nairobi, Kenya; 2 Biotechnology and Biosafety Research Center, Agricultural Research Corporation, P.O. Box 126, Khartoum, Sudan

**Keywords:** Sweet sorghum, immature embryos, callus induction frequency, 2, 4-D, regeneration

## Abstract

**Background and Objective:**

Regeneration of plant through tissue culture technique is a critical process in transformation of plants. This study sought to establish the effect of genotype and auxin concentration on callus induction from different sweet sorghum genotypes (IESV92008DL, IESV92001DL, IESV92021DL, ICSV700 and ICSV93048).

**Materials and Methods:**

In this study, MS medium supplemented with five levels of the hormone 2,4-D (0, 1, 2, 4 and 6 mg L^-1^) to assess the effect of the hormone 2,4-D on callus induction and regeneration was used.

**Results:**

The highest callus induction frequency was observed at 2 mg L^-1^ 2,4-D for all the genotypes, IESV92008 gave the highest callus induction frequency among all the genotypes at 2 mg L^-1^ 2,4-D. The lowest callus induction frequency was observed 0 and 6 mg L^-1^ for all the genotypes, the two genotypes ICSV700 and IESV92021 were observed to give the lowest callus induction frequency among all the genotypes used in this study. Embryogenic callus induction frequency was observed to be higher at 2 mg L^-1^ 2,4-D and the genotype IESV92008 gave the highest callus induction frequency among all the genotypes used. Induction of shoots was achieved in MS medium supplemented with 3 mg L^-1^ 6BA and 1 mg L^-1^ IAA, the highest regeneration efficiency was obtained from the three genotypes ICSV93048, IESV92008 and IESV92001, respectively.

**Conclusion:**

This study discovers the importance of the auxin 2,4-D on callus induction and regeneration of sweet sorghum and this will help the researcher to develop protocols for transformation of sweet sorghum.

## INTRODUCTION

Low yield is a major factor that affect maize crop in the East and Central Africa region leading to lack of adequate food and repeated spell of hunger leading to malnourishment and related diseases among the population. Alternative demands for maize for animal feed and bio fuel has further alleviated maize demand as source of food due to the better price provided by industrial users. In the East and Central Africa region maize yield is often inadequate due to abiotic stress such as drought, aluminum toxicity or scarcity of nutrients, biotic stress being mainly pests and diseases^1^.

Sweet sorghum has high sugar content and promising biofuel feedstocks due to this, it is propagated via seeds and it has short duration to maturity. It require low water compare to the other cereal crops and has high tolerance to disease and salt which made sweet sorghum suitable for lands that mad for other crops^2^.

Sweet sorghum [*Sorghum bicolor* (L.)] is the only crop providing grain and stem for food and animal feed, also it is used as substrates for the production of sugar, alcohol, syrup and fuel, sweet sorghum has been used for bedding, roofing, fencing and paper^3^. Sweet sorghum has potential as an energy crop because it can be cultivated in almost all temperate and tropical areas^4^. Many studies have been conducted for developing new cultivars with high grain yield and high sugar content^5,6^. However, fermentation process it is important for production of ethanol from sweet sorghum juice. Biofuel is very important as source of energy and there are many studies have been focus on production of ethanol from sweet sorghum by using *Saccharomyces cerevisiae* in batch processes^7,8^. This study sought to establish the effect of genotype and auxin concentration on callus induction from different sweet sorghum genotypes ( IESV92008DL, IESV92001DL, IESV92021DL, ICSV700 and ICSV93048).

## MATERIALS AND METHODS

**Plant materials:** The study was carried out at BecA- -ILRI hub in Nairobi, Kenya. Spikes of 5 sweet sorghum genotypes (IESV92008DL, IESV92001DL, IESV92021DL, ICSV700 and ICSV93048) were harvested 16-17 days after pollination then spikes were washed under running tap water for 30 min and sterilized in 70% analytical ethanol for 3 min. The spikes were then soaked in 2.5% sodium hypochlorite for 1 h before washing with sterile water three times under doubled filter laminar flour. The immature embryos were removed using sterile forceps with sharp scalped blades then sterile immature embryos were used as sources of explants for callus induction and regeneration of sweet sorghum. The experiment were designed in RCBD and 4 replication for each 2,4-D level.

Immature embryos were cultured on callus induction medium CIM containing Murashige and skoog 4.4 mg L^-1^ of MS basal salts and vitamins supplemented with 1 g L^-1^ L-proline, 0.5 g L^-1^ casein hydrolysate, 10 mg L^-1^ vitamin C, 0.5 g L^-1^ MES, 0.2 mg L^-1^ kinetin and 45 g L^-1^ sucrose. The pH of the medium was adjusted to 5.8 with 1M NaOH or 0.1 M HCL. About 2.7 g L^-1^ gelrite was added to solidify the medium. The CIM was sterilized by autoclaving then the medium was poured on 90×25 mm petri dishes. The plates warped with parafilm and covered with aluminium foil after culturing and kept in the dark at 25±1°C for 4 weeks. Callus induction frequency was recorded after 4 weeks of culture on callus induction medium and embryogenic callus induction frequency also calculated after transfer to maturation medium while regeneration efficiency was calculated after 4 week incubation on regeneration medium.

After inducing callus all embryogenic callus were transferred to baby jars containing shoot induction medium containing 4.4 g L^-1^ MS basal salts and vitamins^9^, 30 g L^-1^ sucrose, 500 mg L^-1^ casein hydrolysate, 600 mg L^-1^ L-proline, 2 mg L^-1^ 6-BA, 1mg L^-1^ IAA, 2.5 g L^-1^ activated charcoal and 2.7g L^-1^ gelrite for 14 days to initiate shoots after transferring the shoots to the medium jars were kept in the light at 25±1°C. The number of regenerated plantlets per calli was evaluated. The plantlets were transfer to root induction medium without hormones (15 g L^-1^ sucrose, 2.2 MS basal salt and vitamins and 2.5 g L^-1^ activated charcoal) for inducing roots. The pH of the medium was adjusted to 5.8 with 1 M NaOH or 0.1M HCL. About 2.7 g L^-1^ gelrite was added to solidify the medium then the autoclaved medium was poured on baby jars and used to induce root from regenerated plantlets.

Hardening of plantlets was done in peatmoss in small pots while regeneration of plants was achieved in soil in the glasshouse^10^. The number of regenerated plants was counted in order to compute the regeneration efficiency, it was calculated as the percentage of the number of regenerated shoots compared to the number of calli regenerating atleast one shoot. Each experiment was replicated four times with 100 embryos per replication. Plantlets were maintained in the glass house till they matured.

**Statistical analysis:** Experiments were set in randomized block complete designed, analyses of variance (ANOVA), the type of ANOVA being used for analyzing data was one-way ANOVA and were done by Statview statistical program^11^ ( SAS User guide, SAS, Institute Inc, Cary, NC, USA) to test the statistical significance of differences among explants source and 2,4-D levels. Mean separation was performed using least significance difference (LSD) test at 5% probability level.

## RESULTS

Statistical analysis showed there was distinguish differences in callus induction frequency among all the genotypes when different level of 2,4-D via immature embryos were used. The level of 2,4-D 2mg L^-1^ gave the highest callus induction frequency CIF while the level 0 and 6 mg L^-1^ of 2,4-D gave lowest callus induction frequency among all the genotypes used in this study. The genotype IESV92008 gave the highest CIF while the genotype IESV92021 gave the lowest CIF among all the genotypes (Table 1).

**Table 1:** Callus induction frequency immature embryos

2,4-D levels (mg L^-1^)	IESV92001	IESV92021	Genotype IESV92008	ICSV700	ICSV93046
0	40.00±4.08^a^	0.00±0.00^a^	62.50±2.50^a^	0.00±0.00^a^	47.50±6.92^ab^
1	70.00±9.13^b^	0.00±0.00^a^	72.50±7.50^a^	10.00±5.77^a^	52.50±4.79^a^
2	77.50±2.50^b^	27.50±6.29^b^	92.50±4.79^b^	25.00±2.89^b^	72.50±8.54^ac^
4	77.50±4.79^b^	10.00±7.07^a^	67.50±2.50^a^	12.50±4.79^ab^	60.00±4.08^a^
6	45.00±2.89^a^	5.00±2.89^a^	60.00±7.07^a^	7.50±4.79^a^	52.50±7.50^a^
LSD	16.94	14.06	16.40	13.12	20.67
p-value	0.0006	0.0059	0.0072	0.0177	0.1422

Values are Mean±SE of 4 replicates. Means followed by the same letter in the same column are not significantly different at p≤0.05

For the genotype IESV92021the highest callus induction frequency was obtained at 2 mg L^-1^ 2,4-D level of and it gave 27.50±6.29 and it was significantly higher than the other 2,4-D levels but there were no significant differences between 0, 1, 4 and 6 mg L^-1^ of 2,4-D level.

The genotype IESV92008 was observed to give the highest callus induction among all the genotypes it gave 92.50±4.79 from 2 mg L^-1^ of 2,4-D level it was significantly higher than all the other 2,4-D levels but there were no significant differences between 0, 1, 4 and 6 mg L^-1^ in CIF while the genotype ICSV700 gave the highest callus induction frequency at 2 mg L^-1^ of 2,4-D level and it was observed to be significantly higher than that of all other 2,4-D level but there was no significant differences between 2 and 4 mg L^-1^ of 2,4-D also there were no significant differences observed among all the other 2,4-D levels. The genotype ICSV93046 gave the highest CIF at 2 mg L^-1^ of 2,4-D and there were no significant differences observed between it and the other 2,4-D level except 0 mg L^-1^ of 2,4-D level (Table 1).

The highest embryogenic callus induction frequency ECIF was observed at 2 mg L^-1^ of 2,4-D level for all the genotypes while the lowest ECIF was observed to be lower at 0 and 6 mg L^-1^ of 2,4-D. The genotype IESV92001gave highest embryogenic callus induction frequency at 2 mg L^-1^ of 2,4-D and observed to be 42.50±4.79 it was significantly higher than that of all other 2,4-D levels but there was no significant differences between the levels 1 and 4 mg L^-1^ and it was observed to be 27.50±6.92 and 22.50±4.79, respectively, there were no significant differences observed between the two levels 0 and 6 mg L^-1^ of 2,4-D it was observed to be 12.50±4.79 and 10.00±4.08, respectively (Table 2). While the genotype IESV92021 gave the highest embryogenic callus induction from 2 mg L^-1^ of 2,4-D was observed to be 5.50±2.89 , but there were no significant differences between all 2,4-D level in embryogenic callus induction frequencies.

**Table 2:** Embryogenic callus induction frequency from immature embryos

2,4-D levels (mg L^-1^)	IESV92001	IESV92021	Genotype IESV92008	ICSV700	ICSV93046
0	12.50±4.79^a^	0.00±0.00^a^	25.00±6.46^a^	0.00±0.00^a^	17.50±4.79^a^
1	27.50±6.92^b^	0.00±0.00^a^	37.50±4.79^abc^	0.00±0.00^a^	35.00±2.89^be^
2	42.50±4.79^c^	5.50±2.89^a^	47.50±7.50^bcd^	10.00±4.08^b^	57.50±2.50^c^
4	22.50±4.79^ab^	2.50±2.50^a^	45.00±6.46^bc^	0.00±0.00^a^	30.00±4.08^de^
6	10.00±4.08^a^	2.50±2.50^a^	27.50±6.29^abc^	0.00±0.00^a^	22.50±4.79^ad^
LSD	14.62	6.45	18.71	5.63	9.22
p-value	0.0028	0.4449	0.0772	0.0069	<0.0001

Values are Mean±SE of 4 replicates. Means followed by the same letter in the same column are not significantly different at p≤0.05

The genotype IESV92008 gave the highest callus induction obtained from 2 mg L^-1^ of 2,4-D was found to be 47.50±7.50 and it was significantly higher than that of 0 and 6 mg L^-1^ of 2,4-D it was found to be 25.00±6.46 and 27.50±6.29 but there was no significant differences observed between 2 mg L^-1^ and the remaining levels of 2,4-D but there were significant differences observed between 0 and 4 mg L^-1^ 2 ,4-D ECIF was found to be 25.00±6.46 and 45.00±6.4, respectively (Table 2).

The genotype ICSV700 gave the highest ECIF at 2 mg L^-1^ of 2,4-D it was found to be 10.00±4 and it was significantly higher than the other 2,4-D level but there was no significant differences observed between the other levels of 2,4-D. The highest ECIF for the genotype IESV93046 obtained at 2 mg L^-1^ of 2,4-D it was found to be 57.50±2.50 (Table 2) and was significantly higher than the other 2,4-D level but there was no significant differences between 0 and 4 mg L^-1^ of 2,4-D also there were no significant differences observed between 4 and 6 mg L^-1^ of 2,4-D level (Table 2).

**Fig. 1(a-d):**
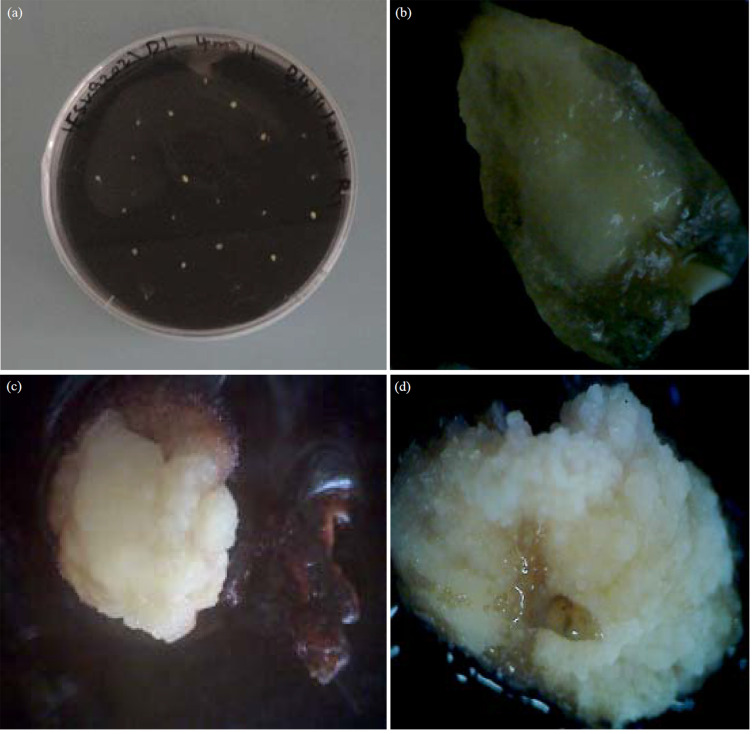
Different calli induced from immature embryos of sweet sorghum, (a) Immature embryos of IESV92021 cultured on CIM medium, (b) Immature embryo of IESV92008, (c) Compact non embryogenic of ICSV 700 induced at 4 mg LG1 of 2,4-D and (d) Friable embryogenic callus of IESV92008 induced at 4 mg LG1 of 2,4-D

Immature embryos of genotypes used in this study (Fig. 1a, b) initiated two types of embryogenic callus types under culture conditions, Type I white, compact and non embryogenic which has been shown in Fig. 1c, while Type II callus white friable and embryogenic callus as has been shown in Fig. 1d.

**Fig. 2:**
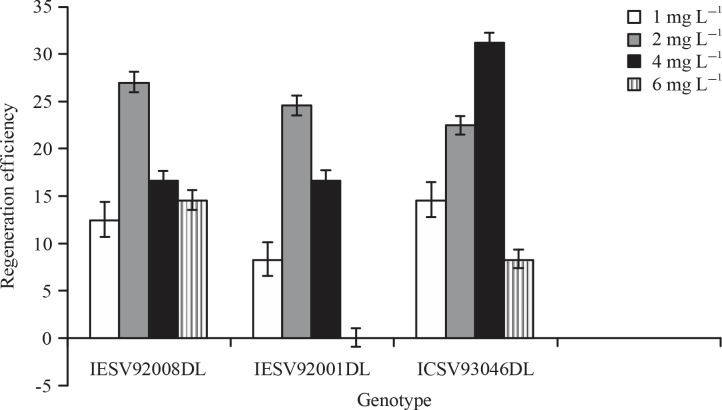
Regeneration efficiency from immature embryos of sweet sorghum using different concentrations of 2,4,D Values are means of 4 replications and vertical bars are standard errors

Callus regeneration efficiency was calculated as the percentage of the number of calli regenerating atleast one shoot over total number of callus tested, However regenerated shoots were higher in the genotype ICSV93046 it gave the highest regeneration efficiency at 4 mg L^-1^ of 2,4-D levels and it was observed to be 31% while 1, 2 and 6 mg L^-1^ of 2,4-D gave regeneration efficiency at 15, 23 and 8% (Fig. 2). The highest regeneration efficiency for genotype IESV92008 was obtained at 2 mg L^-1^ was found to be 27%, the other level of 2,4-D 1, 4 and 6 mg L^-1^ produced regeneration efficiency of 13 , 17 and 15%, respectively. The genotype IESV92001gave the highest regeneration efficiency at 2 mg L^-1^ was found to be 25% while 1 and 4 mg L^-1^ gave regeneration efficiency of 8 and 17%, respectively (Fig. 2).

**Fig. 3(a-d):**
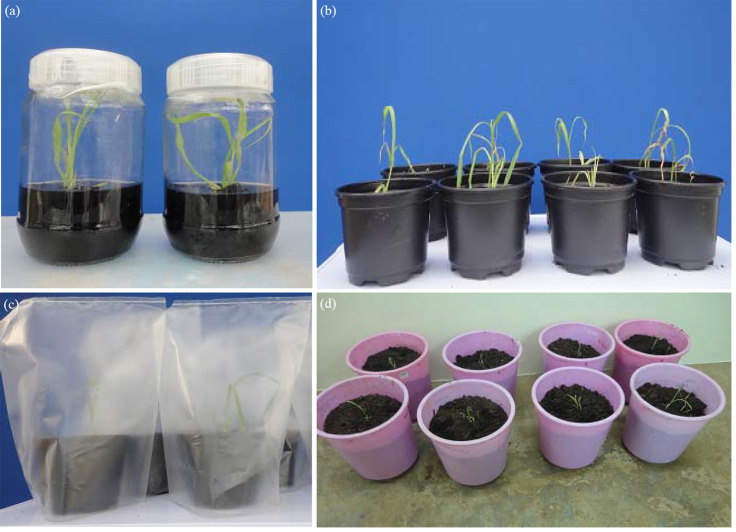
Regeneration of plants from embryogenic callus of sweet sorghum via immature embryos. (a) Multiple shoots induced on regeneration medium, (b) Plantlets transfer to small pots for hardening (c) Hardening of regenerated plantlets at the screen house and (d) Regenerated plants at the screen house

Mature callus transferred to shoot induction media started greening 2-5 days after transfer to light. Regeneration was achieved only in the genotypes IESV92008, IESV92001 and ICSV93046 these genotypes regenerated plants when the callus transfer to shoot induction medium (Fig. 3a).

After 2-3 weeks of culture in shooting or rooting media, plantlets with a well developed root system were transferred to peat moss for glass house conditioning (Fig. 3b). Most of the plantlets survived hardening (Fig. 3c) and were transferred to the soil, plants were able to established well developed root and vegetative growth system (Fig. 3d).

## DISCUSSION

Many factors have been reported to affect tissue culture including tissue culture components such as 2,4-D concentrations, proline, tissue culture conditions and *in vitro* manipulations. Immature embryos they found to give different responses to tissue culture depending on the age of explants and the concentrations of Auxin^12,13^ in this study it was found that callus formation and regeneration was highly stimulated by exposure to 2 and 4 mg L^-1^ of 2,4-D for all the most of the genotypes, addition of the hormone 2,4-D in the culture medium it is important for callus induction from mature and immature embryos of cereal crops^14^.

Immature embryos have been shown to initiate the two types of callus^15^. Type I callus is white, compact and organogenic while Type II is white friable and embryogenic. The type of callus induced has been shown to be dependent on the genotype^16^. Two genes have been implicated in the inheritance of callus induction and plant regeneration. These genes find expression in the middle and basal portion of the scutellum of immature embryo. In case of responsive genotypes, these regions proliferate to form embryogenic callus while in non-embryogenic genotypes, they don’t proliferate^17^.

There are many reports on the use of mature and immature embryos as sources of explants in sorghum tissue culture and transformation^18^, but it is not easy to obtain explants throughout the year but mature seeds are ready available throughout the year. Mature seeds are difficult for tissue culture because of their recalcitrant to regeneration^19^. Sorghum has been reported as one of the most difficult crops for tissue culture and transformation^20^. Transformation of sorghum has been achieved through microprojectile bombardment of immature through zygotic embryos and *Agrobacterium* by using hairy root system through soil bacteria *Agrobacterium rhizogenes*^21^.

## CONCLUSION

It is concluded that in this study protocols for tissue culture and regeneration of sorghum from immature embryos has been optimized successfully. This report shows that it is possible to improve tissue culture conditions for specific varieties by optimizing the compositions of callus induction and plant regeneration media, the genotypes IESV92008 and ICSV93046 are the most promising sweet sorghum genotypes for callus induction regeneration. The optimization of tissue culture protocols of sorghum provides foundation for genetic transformation for improving important traits such as drought and salt tolerant.

## SIGNIFICANCE STATEMENT

This study discovers the importance of the auxin 2,4-D on callus induction and regeneration of sweet sorghum and this will help researchers to develop protocols for transformation of sweet sorghum. This is the first study in East and Central Africa for optimize tissue culture protocol of sweet sorghum.

## Data Availability

All relevant data are within the paper and its supporting information files.
